# Behavioral routines and perceived psychosocial influences associated with perceived academic standing among Moroccan secondary students: a self-regulated learning perspective

**DOI:** 10.1186/s40359-026-04264-4

**Published:** 2026-03-20

**Authors:** Abdelghni El Amoumri

**Affiliations:** Laboratory of Political Studies and Public Law at Sidi Mohamed, Ben Abdellah University, Fez, Morocco

**Keywords:** Time use, Sleep, Digital distraction, Motivation, Family support, Perceived academic standing, Moroccan secondary education, Self-regulated learning

## Abstract

**Background:**

Time use, late-night digital engagement, and psychosocial supports are frequently cited correlates of academic functioning among adolescents, yet evidence from Moroccan secondary education remains limited.

**Methods:**

We conducted two complementary, cross-sectional surveys in public secondary schools in Fez, Morocco. Survey 1 (*N* = 312) assessed nightly routines (bedtime and dominant late-night activities), morning readiness, classroom experiences, and perceived academic standing using a single item. Survey 2 (*N* = 323) assessed perceived academic standing and the perceived influence (no influence to very strong influence) of key psychosocial and contextual factors (e.g., teaching method, family support, sleep, phone/internet use). Analyses were exploratory and performed separately within each survey using descriptive statistics and nonparametric tests; open-ended items were summarized thematically.

**Results:**

In Survey 1, perceived academic standing differed by morning readiness (Kruskal–Wallis H = 24.08, *p* < .001) and grade level (H = 25.21, *p* < .001). Reporting an ability to concentrate in class was associated with higher perceived standing (Mann–Whitney *p* < .001). In Survey 2, teaching method (57% reporting strong/very strong influence), phone/internet use (47%), sleep (45%), and family support (43%) were most frequently rated as strongly influential. Perceived influence of phone/internet use and school environment differed by perceived academic standing category (*p* < .05).

**Conclusions:**

Across two complementary surveys, Moroccan secondary students’ perceived academic standing co-occurred with differences in morning readiness, classroom concentration, and the perceived salience of instructional, family, sleep, and digital-life factors. Findings are descriptive and hypothesis-generating; future work should employ validated multi-item measures, linked designs, and objective grades.

**Supplementary Information:**

The online version contains supplementary material available at 10.1186/s40359-026-04264-4.

## Introduction

### Moroccan secondary education context

Moroccan secondary schooling is a high-stakes phase in which students transition from foundational learning to intensified subject specialization and examination demands. In the baccalaureate years, academic pressure increases as students prepare for high-stakes assessments that shape post-secondary trajectories. Within public secondary schools, students may also encounter crowded classrooms, variable access to learning resources, and heterogeneous instructional quality. At home, study conditions differ widely across households, and adolescents often balance schoolwork with family responsibilities and commuting time. These contextual factors provide an important backdrop for understanding day-to-day learning routines and students’ perceptions of what helps or hinders their academic functioning. International indicators similarly highlight persistent inequality and resource constraints in secondary schooling [1, 2], and local work in Morocco points to motivation patterns and time pressure among secondary students [3, 4].

Adolescents’ daily routines are also increasingly shaped by late-evening screen use and the availability of always-on social communication and entertainment [5]. International research has raised concerns that digital engagement late at night can displace sleep, fragment attention, and reduce morning alertness [6, 7]. However, digital activity can also serve social and emotional functions and may not uniformly predict poorer outcomes; associations can vary by activity type, timing, and individual differences. In Moroccan public-school settings, the interplay between digital life, sleep, and academic routines is likely shaped by local constraints (e.g., shared devices, household schedules, study space) and school-level factors [8].

### Sleep, morning readiness, and classroom functioning

Sleep is a biologically and socially regulated behavior with direct implications for learning. Insufficient or irregular sleep can compromise sustained attention, working memory, and emotional regulation—functions that support classroom learning and exam performance. For secondary students, morning readiness (e.g., feeling alert upon waking, arriving at school with energy) can be viewed as a proximal indicator of the day’s cognitive starting point. In many school systems, including Morocco, core subjects are often scheduled in the first morning periods, making early-day readiness especially relevant. Beyond sleep itself, students’ perceived classroom experiences—such as the ability to concentrate, follow explanations, and remember presented information—capture learning conditions closer to the instructional moment than general routine indicators.

Despite strong theoretical reasons to expect links among sleep-related readiness, attention, and achievement, routine indicators such as bedtime categories may not map straightforwardly onto academic outcomes. Students differ in chronotype, sleep need, and compensatory strategies (e.g., napping, weekend catch-up sleep), and they experience different academic workloads and stress levels. Consequently, descriptive evidence that documents how bedtime timing, late-night activities, morning readiness, and classroom experiences co-occur with students’ perceived standing can generate testable hypotheses for more rigorous measurement and design in future work.

### Self-regulated learning and motivation as interpretive lenses

Self-regulated learning (SRL) refers to how learners plan, monitor, and adapt cognition, motivation, and behavior to reach learning goals, including time and resource management and the regulation of attention and effort [9–12]. Research on time-management practices as part of SRL links them to academic performance and stress regulation [13–17]. Self-determination theory (SDT) complements SRL by emphasizing the quality of motivation and the social conditions that support it (autonomy, competence, and relatedness) in families and classrooms [18–20]. Related motivational perspectives include expectancy-value theory and social cognitive views of motivation [21, 22]. In this study, SRL/SDT are used strictly as interpretive lenses to contextualize descriptive patterns rather than as confirmatory models.

### Psychosocial and contextual influences in the Moroccan setting

Moroccan secondary students operate within a network of psychosocial and contextual influences that can shape academic functioning [23]. Teacher–student relationships, classroom climate, and teaching methods can influence engagement opportunities, perceived competence, and willingness to participate [24, 25]. Peer relationships can provide support and academic collaboration but can also create distraction or social pressure [26]. Family support can take multiple forms, including encouragement, monitoring, provision of study space, and assistance with routines; however, families differ in available resources and time, and students’ responsibilities at home may constrain study time [27, 28].

Students’ perceptions of what influences their academic level provide a valuable window into everyday experiences. Such perceptions do not establish causality, but they can highlight targets for school dialogue and for future research. In under-researched contexts, exploratory descriptive studies can therefore play a legitimate role: mapping patterns, documenting students’ perspectives, and generating hypotheses that can be tested in later confirmatory designs.

### Rationale and aims of the present study

Following reviewer feedback and alignment between the available data and defensible inferences, the current manuscript is deliberately positioned as an exploratory, descriptive contribution based on two complementary surveys. Because the surveys were administered anonymously and cannot be linked at the individual level, they are analyzed separately and interpreted as complementary snapshots rather than a single unified dataset.

Survey 1 examines nightly routines (bedtime timing and dominant late-night activity), morning readiness, and perceived classroom experiences, and explores how these indicators co-occur with a single-item measure of perceived academic standing. Survey 2 examines perceived academic standing and students’ ratings of the strength of influence of key psychosocial and contextual factors (e.g., teaching method, sleep, phone/internet use, family support, and school environment). Together, the two surveys provide descriptive evidence on routines and perceived influences and outline a roadmap for future work that can employ validated instruments, linked designs, and objective achievement outcomes.

Accordingly, the aims are: (1) to describe patterns of nightly routines, morning readiness, and classroom experiences among Moroccan secondary students in Fez; (2) to examine whether these routine and experience indicators co-occur with differences in perceived academic standing within Survey 1; (3) to describe which psychosocial and contextual factors students most frequently rate as strongly influential within Survey 2; and (4) to propose design and measurement steps for future confirmatory studies that can appropriately test integrated SRL/SDT models.

### Contribution of the present study

The contribution of this study is therefore not the confirmation of an explanatory causal model, but the provision of an empirically grounded descriptive map in a local Moroccan setting. The surveys document (a) how students describe their late-night routines and morning readiness and how these co-occur with perceived standing, and (b) which domains students identify as influential for their academic level. By reporting these patterns transparently and acknowledging measurement constraints, the study provides a defensible basis for subsequent instrument development and linkable designs. This contribution is particularly relevant for researchers and practitioners seeking to design context-sensitive interventions and measurement tools for Moroccan public secondary schools.

## Methods

### Study design and setting

This research employed an exploratory, cross-sectional design based on two complementary surveys administered in public secondary schools in Fez, Morocco (March–May 2025). The surveys were designed to capture (a) students’ nightly routines and perceived daytime functioning (Survey 1) and (b) students’ perceived academic standing and perceived influence of psychosocial and contextual factors (Survey 2). Because the two questionnaires were completed anonymously and were not linkable at the individual level, analyses were conducted separately within each survey and interpreted as descriptive and hypothesis-generating.

### Participants and sampling

Survey 1 included *N* = 312 students (174 female, 132 male, 6 missing). Survey 2 included N = 323 students (181 female, 125 male; 17 missing/ambiguous). Students were drawn from the common core, first year baccalaureate, and second year baccalaureate levels. Sampling was pragmatic within participating schools; therefore, findings should not be generalized to Moroccan secondary students nationally. Table 1 presents the demographic characteristics of participants across Survey 1 and Survey 2.

**Table 1:** Sample characteristics (Survey 1 and Survey 2)

Survey	Characteristic	Category	N	%
Survey 1	Gender	Female	174	55.8
Survey 1	Gender	Male	132	42.3
Survey 1	Gender	Missing	6	1.9
Survey 1	Grade level	Second-year baccalaureate	123	39.4
Survey 1	Grade level	First-year baccalaureate	93	29.8
Survey 1	Grade level	Common core	91	29.2
Survey 1	Grade level	Missing	5	1.6
Survey 2	Gender	Female	181	56.0
Survey 2	Gender	Male	125	38.7
Survey 2	Gender	Missing	17	5.3
Survey 2	Grade level	Common core	110	34.1
Survey 2	Grade level	Second-year baccalaureate	100	31.0
Survey 2	Grade level	First-year baccalaureate	99	30.7
Survey 2	Grade level	Missing	14	4.3
Survey 2	Stream	Sciences	247	76.5
Survey 2	Stream	Arts/Literature	50	15.5
Survey 2	Stream	Missing	15	4.6
Survey 2	Stream	Other/Unknown	11	3.4

Percentages are computed within each survey based on all respondents. Some entries were missing or ambiguous (e.g., multi-select responses) and are reported as missing

### Measures

Perceived academic standing was assessed in both surveys using a single self-report item. In Survey 1, students rated recent academic standing on a four-category scale (excluding the ‘prefer not to answer’ option). In Survey 2, students indicated whether their achievement was ‘insufficient’, ‘acceptable’, ‘average’, ‘good’, or ‘very good’.

Survey 1 additionally included categorical items on bedtime timing on school nights, dominant late-night activity, morning readiness, and perceived classroom experiences (multi-select). Survey 2 included single items assessing the perceived influence (no influence to very strong influence) of key factors on academic level, including socioeconomic resources, relationships with teachers and peers, school environment, teaching method, phone/internet use, adequate sleep, and family support. These items capture perceived salience/importance rather than directional valence (helping vs hindering); open-ended questions in both surveys captured students’ explanations and advice.

### Measurement considerations (validity, reliability, and language)

Both surveys were designed as brief, pragmatic questionnaires to minimize classroom burden and preserve anonymity. Accordingly, several constructs were operationalized using single items and coarse categorical indicators. Single-item measures do not permit internal-consistency reliability estimation (e.g., Cronbach’s alpha/omega) or factorial validation within the present dataset; this is treated as a key limitation and motivates the roadmap for validated multi-item measurement outlined in the Discussion.

Items were administered in the language used in participating schools (Arabic/French). Because the items were developed for this exploratory study and were not adapted from an established Moroccan-validated scale, no formal cross-cultural adaptation procedure (e.g., forward/back translation with cognitive interviewing and CFA-based measurement invariance) was performed. To improve face clarity, the survey wording was reviewed with teachers prior to administration, and response options were kept simple and concrete. Future work should employ validated Arabic/French instruments (or a full adaptation and psychometric validation process) and link responses to objective grades. The full item wording and response options were included in the questionnaire materials provided to the journal during the submission process.

### Procedure

Surveys were administered via Google Forms under teacher supervision. Participation was voluntary and anonymous. Students were informed about confidentiality and their right to withdraw. Completion time was approximately 15–20 min.

### Data analysis

Analyses were conducted in IBM SPSS Statistics (v28) and replicated for reporting. Given the ordinal nature of perceived academic standing and categorical predictors, nonparametric tests were used (Kruskal–Wallis and Mann–Whitney U). Effect sizes are reported using epsilon-squared for Kruskal–Wallis and r for Mann–Whitney tests, and are interpreted using common conventions (small ≈ 0.01/0.10, medium ≈ 0.06/0.30, large ≈ 0.14/0.50, respectively) [29]. Because the surveys were anonymous and brief, key covariates (e.g., prior grades, commute duration, and more detailed socioeconomic indicators) were not available; therefore, all inferential analyses are unadjusted bivariate comparisons and are interpreted cautiously. Because the two surveys were not linkable at the individual level, no integrated mediation, moderation, or latent-variable SEM was conducted in this revision [30].

Missing/ambiguous entries were handled by available-case analysis within each test. For Kruskal–Wallis tests, epsilon-squared was computed as (H − k + 1)/(n − k) and is reported as 0 when negative due to sampling variability; for Mann–Whitney tests, r was computed as Z/√N.

Open-ended responses were summarized using a brief content-analytic thematic summary based on frequent keywords and recurrent ideas. This qualitative component is descriptive and is used to contextualize quantitative patterns rather than to provide a standalone, theory-generating qualitative analysis.

### Ethics

Ethical approval and consent procedures are reported in the Declarations section. No personal identifiers or official grades were collected.

## Results

### Survey 1 results

Of the Survey 1 respondents (*N* = 312), perceived academic standing was provided by 253 students; 53 selected ‘prefer not to answer’ and were excluded from inferential analyses. Bedtime timing on school nights clustered between 11 pm and 1 am (57%), while 23% reported sleeping before 11 pm. Nonparametric comparisons examining associations between behavioral predictors and perceived academic standing are summarized in Table 2.

**Table 2:** Survey 1 nonparametric comparisons for perceived academic standing

Predictor	H	P	Epsilon-squared
Bedtime timing (school nights)	7.43	.060	0.018
Dominant late-night activity	8.39	.136	0.014
Morning readiness	24.08	<.001	0.085
Grade level	25.21	<.001	0.093

Bedtime timing (school nights): H = 7.43, *p* =.060, ε2 = 0.018. Dominant late-night activity: H = 8.39, *p* =.136, ε2 = 0.014. Morning readiness: H = 24.08, *p* <.001, ε2 = 0.085. Grade level: H = 25.21, *p* <.001, ε2 = 0.093

Morning readiness showed the largest observed effect size in Survey 1 (ε2 ≈ 0.085). Students reporting being ‘active and ready’ in the morning tended to report higher perceived standing than those reporting severe fatigue or early-period concentration difficulty. Associations between classroom experiences and perceived academic standing in Survey 1 are presented in Table 3.

**Table 3:** Survey 1 classroom experiences (multi-select) and perceived academic standing

Item	Selected n	Mean (sel.)	Not sel	Mean (not)	U	p	R
I can concentrate well	47	2.43	206	1.92	6378.0	<.001	0.213
I find it difficult to remember presented information	67	1.84	186	2.08	5130.0	.014	0.135
My thoughts are very scattered	78	1.90	175	2.07	5867.0	.040	0.112
I feel sleepy and try to resist	120	1.97	133	2.06	7431.0	.277	0.059
I feel tense or irritable easily	42	1.88	211	2.04	4053.5	.316	0.055

Classroom experience items were multi-select; for each item, Mann–Whitney tests compare students who selected vs did not select the option

Reporting the ability to concentrate in class was associated with higher perceived standing (*p* < 0.001). By contrast, reporting difficulty remembering presented information and reporting scattered thoughts were associated with lower perceived standing (*p* < 0.05).

### Survey 1 open-ended responses

Students’ open-ended descriptions of the perceived benefits of staying up late most frequently referenced entertainment (watching films/series, gaming), social communication, and a perceived sense of quiet time. Common advice to peers emphasized sleeping earlier, reducing phone use, and organizing study time. Frequently occurring keywords included: watching, time, benefit, sleep, films/series, insomnia, studying, concentration, and phone.

### Survey 2 results

Survey 2 included *N* = 323 students; perceived academic standing was available for 308 students after excluding missing/ambiguous entries. The distribution was: insufficient (*n* = 14), acceptable (*n* = 55), average (*n* = 116), good (*n* = 103), and very good (*n* = 20).

Students rated the perceived influence of several factors on their academic level. Because these items capture perceived salience rather than directional causality, results are interpreted descriptively. Descriptive statistics for the perceived influence of key factors on academic standing in Survey 2 are shown in Table 4; they do not indicate whether each factor is helping or hindering.

**Table 4:** Survey 2 perceived influence of key factors

**Factor**	**Valid n**	**Mean influence (0–4)**	% strong/very strong (≥ 3)	**Rank (by % strong)**
Teaching method	305	2.53	57.0%	1
Teacher-student relationship	302	1.93	48.7%	2
Phone/Internet use	304	2.28	47.4%	3
Adequate sleep	306	2.23	45.4%	4
Family support	302	2.18	43.4%	5
School environment	305	1.86	34.8%	6
Peer relationship	306	1.83	32.0%	7
Family socioeconomic status	304	1.27	25.7%	8

Influence scale: 0 = no influence, 1 = weak, 2 = moderate, 3 = strong, 4 = very strong. “Strong/very strong” corresponds to responses ≥ 3

To examine whether perceived influence differed by students’ perceived academic standing, nonparametric comparisons were conducted. Results of these comparisons are summarized in Table 5.

**Table 5:** Survey 2 differences in perceived influence by perceived academic standing

Factor	H	P	Epsilon-squared
Phone/Internet use	17.59	.002	0.045
School environment	12.97	.011	0.030
Family socioeconomic status	7.55	.109	0.012
Teaching method	7.00	.136	0.010
Family support	6.93	.140	0.010
Adequate sleep	5.96	.203	0.006
Peer relationship	3.82	.431	0.000
Teacher-student relationship	2.36	.669	0.000

As shown in Table 5, perceived influence differed by perceived academic standing for Phone/Internet use (H = 17.59, *p* = 0.0015, ε2 = 0.045); School environment (H = 12.97, *p* = 0.0114, ε2 = 0.030). Other factors showed no clear differences across standing categories (Family socioeconomic status (H = 7.55, *p* = 0.1094, ε2 = 0.012); Teaching method (H = 7.00, *p* = 0.1361, ε2 = 0.010); Family support (H = 6.93, *p* = 0.1395, ε2 = 0.010); Adequate sleep (H = 5.96, *p* = 0.2025, ε2 = 0.006); Peer relationship (H = 3.82, *p* = 0.4311, ε2 = 0.000); Teacher-student relationship (H = 2.36, *p* = 0.6690, ε2 = 0.000)).

Perceived influence of phone/internet use and school environment differed across perceived academic standing categories (*p* < 0.05), suggesting heterogeneity in how students at different perceived standing levels interpret these influences.

### Survey 2 open-ended responses

In open-ended responses on negative influences, students frequently mentioned phone use, poor time organization, concentration difficulties, teacher-related issues, and sleep problems. Frequently occurring keywords included: phone, lack of study time, time, classroom, school, teachers, academic work, and problems.

## Discussion

### Principal findings

This revision reports two complementary cross-sectional surveys of public secondary students in Fez. Across both surveys, students’ perceived academic standing co-occurred with indicators plausibly relevant for learning in daily school life: morning readiness and perceived classroom concentration (Survey 1), and the perceived salience of teaching practices, sleep, phone/internet use, and family support (Survey 2). Because the surveys are not linkable at the individual level and perceived standing was measured with a single item, the findings are interpreted as descriptive and hypothesis-generating rather than as evidence of causal mechanisms.

### Survey 1: morning readiness and classroom functioning

Within Survey 1, morning readiness showed the clearest association with perceived academic standing. Students reporting waking up ‘active and ready’ tended to report higher standing than those reporting severe fatigue or early-period concentration difficulty. This pattern is compatible with the interpretation that readiness is a proximal condition for attention and effort during the school day. From an SRL perspective, readiness may reflect a combination of regulation in the previous evening (sleep and screen routines), anticipation of academic demands, and emotional readiness to engage with instruction [9, 10]. From an SDT perspective, readiness may also be shaped by perceived competence and classroom climate: students who expect to succeed or feel supported may approach the day with greater energy [18–20]. Achievement emotions may also contribute to readiness and engagement in class [31].

The multi-select classroom experiences add further context. Students who reported an ability to concentrate in class tended to report higher perceived standing, whereas reports of scattered thoughts or difficulty remembering presented information co-occurred with lower standing. These experience-near indicators may be especially relevant because they capture what students believe happens during learning episodes rather than only what happens before school. In practical terms, the patterns highlight that perceived academic standing is intertwined with everyday cognitive functioning—alertness and attention—within the classroom.

Bedtime timing and dominant late-night activity showed weaker or non-significant differences in perceived standing in this exploratory dataset. This null pattern should not be interpreted as evidence that sleep timing or late-night digital activity is inconsequential. A more cautious interpretation is that broad categorical indicators (e.g., bedtime ranges) are not sufficiently sensitive and that unmeasured factors—sleep duration, sleep quality, variability across weekdays and weekends, chronotype, and academic workload—may moderate associations. It is also plausible that students compensate for late bedtimes in ways not captured here (e.g., napping or earlier sleep on non-school nights), or that the consequences of late-night activity are expressed primarily through morning readiness, which did show clear differences. Individual differences such as personality traits may also be relevant and were not measured in this study [32, 33].

### Survey 2: perceived salience of psychosocial and contextual influences

Survey 2 complements the routine-focused snapshot by asking students to rate how strongly several factors influence their academic level. Teaching method emerged as the factor most frequently rated as strongly influential, followed by phone/internet use, adequate sleep, and family support. This ordering suggests that students do not frame academic functioning purely as an individual self-control issue; they also emphasize instructional and relational environments. In Moroccan public-school contexts, perceptions of teaching method may reflect clarity of explanations, lesson pacing, opportunities for practice, feedback quality, and classroom management—all of which shape engagement opportunities and perceived competence. Work on learners’ expectations during educational transitions also highlights the importance of aligning teaching practices with students’ needs [34].

Students’ emphasis on phone/internet use and sleep aligns with broader concerns about attention fragmentation and sleep displacement [6–8]. In the present data, these are captured as students’ perceptions rather than as objectively measured exposures, but they nonetheless highlight domains that students themselves experience as salient. Family support also ranked highly, suggesting that encouragement, monitoring, and the provision of study structure may help students navigate school demands [27, 28]. At the same time, socioeconomic status and broader structural determinants may still shape objective outcomes even if they are not the most vivid influences in students’ day-to-day interpretations.

Differences across perceived academic standing categories in the perceived influence of phone/internet use and school environment suggest heterogeneity in how students interpret these domains. Students who perceive themselves as struggling may experience digital distractions or environmental constraints as more disruptive, or may be more sensitive to barriers in classroom climate and school resources. Alternatively, higher-performing students may be more aware of opportunity costs and self-regulation demands. Because the influence items were not directionally valenced (helping vs. hindering), these interpretations remain tentative and motivate more precise item design.

### Complementary insights and cautious integration

Although the two surveys cannot be statistically integrated, their convergence can support a coherent descriptive account. Survey 1 highlights proximate, experience-near correlates—morning readiness and perceived classroom concentration—that co-occur with perceived standing. Survey 2 highlights the domains students identify as influential—teaching practices, sleep, digital life, and family support. Taken together, the results support a plausible hypothesis that students’ readiness and attentional functioning in class reflect a broader ecology of routines and supports. This hypothesis is consistent with SRL and SDT as interpretive frameworks, but it requires a single-sample, linkable design with validated measures and objective grades to test [9, 10, 18, 19].

### Strengths and limitations

The study contributes school-based evidence from an under-researched context with relatively large samples and transparent reporting of an exploratory analytic strategy. However, interpretation is constrained by several limitations: (1) the two surveys were independent and not linkable at the individual level; (2) key constructs were measured using single items and categorical indicators rather than validated multi-item scales; (3) perceived academic standing was self-reported rather than verified against official grades; and (4) cross-sectional self-report data cannot establish temporal ordering or causal pathways.

A further limitation concerns the interpretability of the Survey 2 ‘perceived influence’ items. Because students were not asked to specify whether each factor affected them positively or negatively, the same response category (e.g., ‘strong influence’) may reflect very different experiences (for example, strong positive influence of supportive teaching versus strong negative influence of excessive phone use). Future questionnaires should explicitly separate facilitating from hindering influences or use clearly valenced statements with consistent directionality. Finally, the open-ended summaries in this revision are intentionally light and should be strengthened in future research through systematic qualitative coding and triangulation.

### Implications and future research

Because the present evidence is exploratory and based on single-item/categorical indicators, implications are framed as hypothesis-generating. Across both surveys, students’ perceived academic standing co-occurred with morning readiness and classroom concentration (Survey 1), and students most often rated teaching method, phone/internet use, sleep, and family support as strongly influential (Survey 2). These descriptive patterns suggest that feasible school dialogue and small-scale pilots could focus on early-day readiness and attention (e.g., sleep-routine support, realistic strategies for evening screen use), alongside instructional practices that enhance clarity and structured engagement.

For future research, we recommend (a) validated multi-item measurement with transparent translation/adaptation procedures, (b) linkable designs that connect survey responses to objective achievement indicators (e.g., semester marks/exam results) where feasible and ethical, and (c) designs that account for clustering within classes/schools. Longitudinal or repeated-measures approaches would enable stronger tests of temporal ordering and, where appropriate, mediation/moderation models (e.g., conditional process approaches) [30].

## Conclusions

Using two complementary surveys in Fez, Moroccan secondary students’ perceived academic standing co-occurred with differences in morning readiness and classroom concentration and with strong perceived influence of teaching practices, family support, sleep, and digital-life factors. These findings are exploratory and context-specific. Stronger evidence will require validated instruments, linked designs, and objective performance indicators.

## Supplementary Information

Supplementary Material 1.

Supplementary Material 2.

Supplementary Material 3.

Supplementary Material 4.

## Data Availability

The datasets generated and analyzed during the current study are not publicly available due to confidentiality agreements with the participants but may be available from the corresponding author upon reasonable request.

## Abbreviations

SRL: Self-Regulated Learning
SDT: Self-Determination Theory
