# Supplementary material for: Behavioral routines and perceived psychosocial influences associated with perceived academic standing among Moroccan secondary students: a self-regulated learning perspective
Source: BMC Psychol. 2026 Mar 20;14:623. doi: 10.1186/s40359-026-04264-4 (PMC13126882; doi:10.1186/s40359-026-04264-4)
Supplement: Supplementary file 1 — Supplementary Material 1. [file 40359_2026_4264_MOESM1_ESM.pdf]

Questionnaire: Factors Influencing the Quality of Academic Achievement

English translation of the Arabic questionnaire (Google Forms)

In the name of Allah, the Most Gracious, the Most Merciful.

Dear students,
This questionnaire aims to study the factors that influence the quality of academic achievement among upper secondary school students, by analyzing different factors that may have a positive or negative effect on your academic results.

These factors include personal, social, and environmental factors, as well as factors related to the curriculum and the teaching method. The goal of this study is to develop a deeper understanding of these factors so that appropriate measures can be taken to improve the learning environment and ensure better academic achievement for all students.

We appreciate your time and effort in participating, and we assure you that all responses will remain confidential and will be used only for scientific research purposes. Please answer honestly and objectively, and feel free to provide any remarks or suggestions you may have to improve the quality of education in our school.

Thank you for your cooperation.

Please place an "X" in the appropriate box or write your answer in the space provided.

General Information

1. Gender

Select one answer.

☐ Male

☐ Female

2. Grade level

Select one answer.

☐ Common Core

☐ First-Year Baccalaureate

☐ Second-Year Baccalaureate

3. Academic stream

Select one answer.

☐ Arts/Literature

☐ Sciences

☐ Mathematics Sciences

4. Do you think your academic achievement is good?

Select one answer.

☐ Very good

☐ Good

☐ Average

☐ Fair

☐ Insufficient

Factors Influencing Academic Achievement

Please indicate the degree to which the following factors affect your academic level:

5. Family socioeconomic status

Select one answer.

☐ Does not affect

☐ Affects a little

☐ Affects a lot

☐ Affects very strongly

6. Relationship with teachers

Select one answer.

☐ Does not affect

☐ Affects a little

☐ Affects a lot

☐ Affects very strongly

7. Relationship with classmates

Select one answer.

☐ Does not affect

☐ Affects

☐ Affects a lot

☐ Affects very strongly

8. School environment (space, equipment, etc.)

Select one answer.

☐ Does not affect

☐ Affects

☐ Affects a lot

☐ Affects very strongly

9. Teaching method

Select one answer.

☐ Does not affect

☐ Affects

☐ Affects a lot

☐ Affects very strongly

10. Private tutoring

Select one answer.

☐ Does not affect

☐ Affects

☐ Affects a lot

☐ Affects very strongly

11. Use of phone and internet

Select one answer.

☐ Does not affect

☐ Affects

☐ Affects a lot

☐ Affects very strongly

12. Adequate sleep

Select one answer.

☐ Does not affect

☐ Affects

☐ Affects a lot

☐ Affects very strongly

13. Family support

Select one answer.

☐ Does not affect

☐ Affects

☐ Affects a lot

☐ Affects very strongly

14. Personal motivation and self-drive

Select one answer.

☐ Does not affect

☐ Affects

☐ Affects a lot

☐ Affects very strongly

Open-ended Questions

15. What factors do you think negatively affect your academic level?

Answer:

16. What could improve the quality of your academic achievement?

Answer:

17. Do you have suggestions to improve learning conditions inside the school?

Answer:
