# Supplementary material for: Behavioral routines and perceived psychosocial influences associated with perceived academic standing among Moroccan secondary students: a self-regulated learning perspective
Source: BMC Psychol. 2026 Mar 20;14:623. doi: 10.1186/s40359-026-04264-4 (PMC13126882; doi:10.1186/s40359-026-04264-4)
Supplement: Supplementary file 2 — Supplementary Material 2. [file 40359_2026_4264_MOESM2_ESM.pdf]

Questionnaire: Time Management and Daily Challenges of Upper Secondary Students

English translation of the Arabic questionnaire (Google Forms)

Dear student,
Please participate in this questionnaire, which aims to study the relationship between staying up late and academic achievement among upper secondary school students. All data will be handled with complete confidentiality and for research purposes only. Thank you for your time and cooperation.

Section 1: General Information

1. Gender

Select one answer.

☐ Male

☐ Female

2. Grade level

Select one answer.

☐ Common Core

☐ First-Year Baccalaureate

☐ Second-Year Baccalaureate

3. How would you describe your academic performance in general during the recent period?

Select one answer.

☐ I consider it excellent compared to my classmates

☐ Very good, and I feel satisfied with it

☐ Acceptable, and I can improve it

☐ I face some difficulties in keeping up

☐ I prefer not to answer

Section 2: Your Evening Habits and Activities

4. On school nights (Sunday to Friday), what time do you usually go to sleep?

Select one answer.

☐ Before 11:00 PM

☐ Between 11:00 PM and 1:00 AM

☐ Between 1:00 AM and 3:00 AM

☐ After 3:00 AM

5. What activity takes up most of your time late at night? (Choose the most dominant activity)

Select one answer.

☐ Chatting and communicating with friends

☐ Browsing social media

☐ Playing electronic games

☐ Watching series or movies

☐ Studying and preparing lessons

☐ Other

6. If you stay up late for other reasons, please specify:

Answer:

Section 3: Your School Day and Challenges

7. How do you feel when you wake up to go to school in the morning?

Select one answer.

☐ Energetic and ready to start the day

☐ I feel somewhat tired, but I can follow the lessons

☐ I feel very tired and find it difficult to concentrate from the first class

☐ I have difficulty waking up, which may make me late or absent

8. What happens to you during lessons, especially in the morning? (You can choose more than one)

Select all that apply.

☐ I can concentrate well

☐ My thoughts are very scattered

☐ I feel sleepy and try to resist

☐ I find it difficult to remember the information presented

☐ I feel tense or easily irritated

Section 4: Your Opinions and Suggestions

9. In your own words, what is the most important benefit or enjoyment you find in staying up late?

Answer:

10. If you wanted to advise a classmate to improve their academic experience, what is the first advice you would give?

Answer:

Thank you for your valuable contribution.
