# Supplementary material for: Behavioral routines and perceived psychosocial influences associated with perceived academic standing among Moroccan secondary students: a self-regulated learning perspective
Source: BMC Psychol. 2026 Mar 20;14:623. doi: 10.1186/s40359-026-04264-4 (PMC13126882; doi:10.1186/s40359-026-04264-4)
Supplement: Supplementary file 3 — Supplementary Material 3. [file 40359_2026_4264_MOESM3_ESM.pdf]

Questionnaire on Factors Influencing Academic Achievement among Secondary School Students

In the name of Allah, the Most Gracious, the Most Merciful

Dear Students,

This questionnaire aims to examine the factors that influence the quality of academic achievement among secondary school students. It seeks to analyze various personal, social, environmental, and instructional elements that may positively or negatively affect your academic performance.

The purpose of this study is to gain a deeper understanding of these factors in order to improve the learning environment and ensure better educational outcomes for all students.

We appreciate your time and effort in participating in this research. Please note that all responses will remain confidential and used solely for academic purposes. Kindly answer sincerely and objectively, and feel free to share any comments or suggestions that may help improve the quality of education at your institution.

Thank you for your cooperation.

Please mark (X) in the appropriate box or provide your answer in the designated space.

GENERAL INFORMATION

1. Gender:
   [ ] Male   [ ] Female

2. Grade Level:
   [ ] Common Core   [ ] 1st Year Baccalaureate   [ ] 2nd Year Baccalaureate

3. Field of Study:
   [ ] Literature   [ ] Sciences   [ ] Mathematics

4. How do you evaluate your academic performance?

[ ] Very Good [ ] Good [ ] Average [ ] Fair [ ] Weak

FACTORS INFLUENCING ACADEMIC ACHIEVEMENT

Please indicate how strongly each of the following factors affects your academic performance:

1. Family’s economic level
2. Relationship with teachers
3. Relationship with classmates
4. School environment (space, facilities, etc.)
5. Teaching methods
6. Private tutoring
7. Use of phone and internet
8. Sufficient sleep
9. Family support
10. Motivation and self-drive

Each item: [ ] Slight Effect [ ] Moderate Effect [ ] Strong Effect [ ] No Effect

OPEN-ENDED QUESTIONS

11. What factors do you believe negatively affect your academic performance?

....

12. What could improve the quality of your academic achievement?

....

13. Do you have suggestions to improve learning conditions at your school?

....

End of Questionnaire — All responses are confidential and used solely for research purposes.
