# Supplementary material for: Behavioral routines and perceived psychosocial influences associated with perceived academic standing among Moroccan secondary students: a self-regulated learning perspective
Source: BMC Psychol. 2026 Mar 20;14:623. doi: 10.1186/s40359-026-04264-4 (PMC13126882; doi:10.1186/s40359-026-04264-4)
Supplement: Supplementary file 4 — Supplementary Material 4. [file 40359_2026_4264_MOESM4_ESM.pdf]

Your Opinion Matters: A Survey on Time Management and Daily Challenges for High School Students

Dear Student,

We kindly ask you to participate in this survey, which aims to study the relationship between staying up late and academic achievement among secondary school students.

All data will be treated confidentially and used for research purposes only. Your responses will help us better understand how students' daily habits, such as time management and sleep patterns, influence academic success.

Thank you for your time and cooperation.

SECTION ONE: GENERAL INFORMATION

1. Sex:
   [ ] Male   [ ] Female

2. Academic Level:
   [ ] Common Core   [ ] 1st Year Baccalaureate   [ ] 2nd Year Baccalaureate

SECTION TWO: DAILY HABITS AND TIME MANAGEMENT

3. How many hours do you usually sleep at night?
   [ ] Less than 5 hours   [ ] 5–6 hours   [ ] 7–8 hours   [ ] More than 8 hours

4. How often do you stay up late (after midnight) during school days?
   [ ] Never   [ ] Rarely   [ ] Sometimes   [ ] Often   [ ] Always

5. How often do you feel tired or sleepy during morning classes?
   [ ] Never   [ ] Rarely   [ ] Sometimes   [ ] Often   [ ] Always

6. How would you rate your ability to manage your study time?

[ ] Excellent [ ] Good [ ] Average [ ] Poor

SECTION THREE: ACADEMIC PERCEPTION

7. Do you think staying up late affects your academic performance?

[ ] Not at all [ ] Slightly [ ] Moderately [ ] Strongly

8. What are the main reasons you stay up late?

[ ] Studying [ ] Internet/Phone Use [ ] Social Activities [ ] Other: ___________

9. What do you think could help you improve your time management?

____

10. Any suggestions for improving students' daily routines at school?

____

End of Survey — All responses are confidential and used solely for research purposes.
